# Chronic Chylothorax in Childhood: Clinical Complexity and Management Challenges—A Case Report and Literature Overview

**DOI:** 10.3390/diagnostics16111614

**Published:** 2026-05-25

**Authors:** Teofana-Otilia Bizerea-Moga, Cristina-Loredana Pantea, Giorgiana Flavia Brad, Otilia Mărginean, Tudor-Voicu Moga

**Affiliations:** 1 Department XI of Pediatrics-1st Pediatric Discipline, Center for Research on Growth and Developmental Disorders in Children, “Victor Babes” University of Medicine and Pharmacy Timisoara, Eftimie Murgu Sq No. 2, 300041 Timisoara, Romania; bizerea.teofana@umft.ro (T.-O.B.-M.); brad.giorgiana@umft.ro (G.F.B.); marginean.otilia@umft.ro (O.M.); 2 3rd Pediatric Clinic from Clinical Emergency Hospital for Children “Louis Turcanu”, Iosif Nemoianu 2, 300011 Timisoara, Romania; 3 Regional Center of Medical Genetics Timis, Clinical Emergency Hospital for Children “Louis Turcanu”, 300011 Timisoara, Romania; 4 Doctoral School, “Victor Babes” University of Medicine and Pharmacy, Eftimie Murgu Sq No. 2, 300041 Timisoara, Romania; 5 Department VII of Internal Medicine-Gastroenterology Discipline, Advanced Regional Research Center in Gastroenterology and Hepatology, “Victor Babes” University of Medicine and Pharmacy Timisoara, Eftimie Murgu Sq No. 2, 300041 Timisoara, Romania; moga.tudor@umft.ro; 6 Gastroenterology and Hepatology Clinic,“Pius Brînzeu” County Emergency Clinical Hospital, Liviu Rebreanu No 156, 300723 Timisoara, Romania

**Keywords:** chylothorax, octreotide, drainage, pediatric pleural effusion

## Abstract

**Background and Clinical Significance:** Pleural effusion results from an imbalance between fluid production and lymphatic drainage within the pleural space and may arise from a wide range of conditions. Chylothorax, characterized by the accumulation of chyle in the pleural cavity, is an uncommon cause of pleural effusion in the pediatric population. Although octreotide has been widely used in neonatal postoperative chylothorax, evidence supporting its role in acquired chylothorax beyond infancy remains limited, and dosing strategies are not standardized. This report describes the management of massive chylothorax in an adolescent patient. **Case Presentation:** A 13-year-old girl presented with fatigue, decreased exercise tolerance, vertigo, and diaphoresis and was diagnosed with massive right-sided chylothorax based on imaging and pleural fluid analysis. Treatment consisted of pleural drainage, dietary measures, and continuous intravenous octreotide administered within a multidisciplinary care framework. Chylous drainage declined progressively, with complete resolution of the effusion achieved without surgical intervention. **Conclusions:** Chylothorax in older children and adolescents may present with nonspecific symptoms and requires a high index of clinical suspicion. Early diagnosis, appropriate drainage, and individualized therapy are essential for favorable outcomes. Continuous octreotide infusion may represent an effective adjunctive treatment in selected pediatric cases, but further studies are needed to define optimal dosing and treatment duration.

## 1. Introduction—Literature Review

Owing its name to the Greek term “chylos,” meaning “juice,” chyle is a milky bodily fluid that originates in the intestine during fat absorption. It essentially consists of lymphatic fluid and chylomicrons, which are composed of long-chain triglycerides, phospholipids, cholesterol, and cholesterol esters. These lipid particles are formed in the jejunal mucosa and transported through the lymphatic system. Biochemically, it is an alkaline fluid with high cellularity (>1000 cells/µL), predominantly lymphocytes, and triglyceride concentrations exceeding 100 mg/dL. Additional components include electrolytes, plasma proteins, and glucose [1,2].

Chyle is transported from the gastrointestinal tract to the systemic venous circulation via the thoracic duct, which drains lymph from the abdomen, lower extremities, and left hemithorax before emptying into the junction of the left subclavian and internal jugular veins [3]. When the thoracic duct is injured, chyle leaks into surrounding structures, and accumulation within the pleural space results in chylothorax. In the postoperative setting, chylothorax most commonly occurs as a complication of thoracic or cardiac surgery, due to inadvertent injury to the thoracic duct or its tributaries during the procedure [4].

### 1.1. Pathogenesis, Etiology and Classification of Chylothorax

According to the underlying mechanism of development, chylothorax can be divided into traumatic, non-traumatic, and idiopathic forms [4,5,6,7,8,9].

Non-traumatic causes in the pediatric population differ from those in adults. While malignancy—particularly lymphoma—predominates in adult non-traumatic chylothorax, congenital lymphatic malformations (such as lymphangiomatosis and generalized lymphatic anomaly) [10,11,12], chromosomal abnormalities (e.g., Turner syndrome and Noonan syndrome), and congenital heart disease are more frequently implicated in children. Congenital chylothorax, often presenting in the neonatal period, may be associated with lymphatic dysplasia or occur as an isolated finding. In approximately 9% of cases across all age groups, no identifiable cause is found, and the condition is classified as idiopathic.

In children and neonates, there are significant differences from adults that should be taken into consideration when analyzing the etiological profile of chylothorax. In the pediatric population, the most common cause is iatrogenic injury during congenital heart disease surgery (e.g., Fontan, Glenn, Norwood, and Blalock-Taussig procedures), with reported incidences ranging from 0.5% to 6.6% of cardiothoracic operations. Other surgical causes include esophageal and mediastinal procedures, as well as thoracic duct injury related to central venous catheterization. Traumatic, most often iatrogenic, chylothorax in children is commonly associated with surgery for congenital heart disease, esophageal malformations, and inborn vascular defects. In neonates, traumatic forms may also be the result of birth-related injury, resuscitation maneuvers, or surgical correction of complex congenital anomalies. Non-surgical traumatic chylothorax is rare in children and may result from birth trauma, forceful coughing or vomiting, or non-accidental injury [8,13,14,15,16,17,18]. In infants and young children, non-traumatic chylothorax also reflects developmental abnormalities of the lymphatic system, such as thoracic duct agenesis, lymphatic dysplasia, lymphangiomatosis, and pulmonary lymphangiectasia. These conditions may occur in association with genetic syndromes such as Down, Turner, and Noonan syndromes, or with disorders characterized by elevated central venous pressure, such as superior vena cava thrombosis and post-Fontan physiology. Congenital chylothorax, often presenting in the neonatal period, may be associated with lymphatic dysplasia or occur as an isolated finding. Pediatric-specific etiologies also include mediastinal and childhood-specific tumors such as neuroblastoma, teratoma, and Wilms tumor. Although rare, infectious and inflammatory diseases such as tuberculosis, histoplasmosis, sarcoidosis, staphylococcal discitis, and Henoch-Schönlein purpura can also ultimately lead to secondary chylothorax [4]. Together, these factors underscore the need for an age-specific approach to chylothorax to ensure accurate diagnosis and appropriate management [13,19].

In approximately 9% of cases across all age groups, no identifiable cause is found, and the condition is classified as idiopathic [20]. Idiopathic chylothorax is diagnosed when, despite a comprehensive evaluation, no traumatic or non-traumatic causes can be established. Even though it is overall less frequently encountered, this form is described more often in neonates and young children, possibly reflecting subtle or unrecognized lymphatic abnormalities in this age category [5,21,22].

Chronic chylothorax is defined as persistence beyond four weeks of conservative management or failure to respond to conservative therapy within two weeks with output >10 mL/kg/day [4,23].

### 1.2. Clinical Presentation

Chylothorax presents with clinical features similar to other pleural effusions and the severity of the symptoms is determined by the amount and rate of chyle accumulation in the pleural space. If the accumulation occurs slowly, as it does in the first stages of non-traumatic chylothorax cases, patients may remain asymptomatic. In contrast, when there is a sudden leakage of chyle following traumatic injury to the thoracic duct, this can rapidly lead to acute respiratory distress and circulatory instability [4,22,23,24,25].

Respiratory complaints, such as cough and dyspnea, are frequent and because chyle is not irritating to the pleura, pleuritic pain and fever are uncommon. Prolonged chyle drainage results in nutritional depletion, electrolyte imbalance, and progressive weight loss. In addition, continuous loss of lymphocytes and immunoglobulins may induce secondary immunodeficiency, increasing susceptibility to infections; although pleural infection itself is unusual due to the bacteriostatic nature of chyle [4,22,23,24,25].

Beyond the postoperative context, chylothorax in older children and adolescents may present insidiously with nonspecific symptoms such as fatigue, decreased exercise tolerance, vertigo, and diaphoresis, further delaying clinical recognition. In such cases, a high index of suspicion is required, as the absence of pathognomonic features and the overlap with more common causes of pleural effusion can lead to diagnostic delay and potentially unfavorable outcomes if left untreated [4].

### 1.3. Imaging Assessment of Chylothorax

For nontraumatic or unknown etiology chylothorax, chest radiographs are useful to confirm the presence and laterality of pleural effusion, and to monitor support lines and tubes, though they cannot distinguish chylothorax from other effusion types [4,9]. Ultrasound can guide thoracentesis for pleural fluid analysis, which remains the diagnostic gold standard (triglycerides > 110 mg/dL). CT imaging plays a crucial role in identifying underlying causes, particularly in nontraumatic chylothorax. CT scan of the chest with contrast can detect mediastinal masses, lymphadenopathy, thoracic malignancies, vascular anomalies, and congenital heart disease—all important etiologies in pediatric patients [4]. When further evaluation is warranted, lymphangiography is helpful if a minimally invasive treatment approach is desired; it can precisely define the leak location and offer therapeutic benefit, but it is not available in all the centers [9]. Pediatric-specific considerations include the high prevalence of congenital heart disease as an underlying etiology [14].

### 1.4. Treatment

The management of pediatric chylothorax is complex. It requires a multidisciplinary approach and is based on a stepwise strategy that takes into account all determinants of the disease. When contouring the treatment plan, the most important factors to consider are the severity of the effusion, the child’s respiratory and metabolic condition, and the underlying pathophysiological mechanism. Until all these factors are clarified, initial care focuses on clinical stabilization of the patient, achieved by evacuation of the pleural fluid when respiratory compromise is present, and parallel confirmation of the diagnosis. The next step in the management of chylothorax is nutritional therapy, which is introduced early to reduce lymphatic fat flow and chyle production. Depending on the patient’s age, special dietary products can be used, such as low-fat diets supplemented with medium-chain triglycerides in children and specially prepared fat-free human milk for newborns and infants. If the leakage of chyle and its secondary buildup in the pleural space is not controlled sufficiently by the dietary regimen alone, then pharmacological agents, such as somatostatin or its analogue, octreotide, may be introduced in the treatment protocol to further decrease lymphatic output [4,5,18,26,27,28].

During treatment, the patient should be closely monitored. The child’s growth, electrolyte balance, and immunological profile should be monitored to avoid prolonged loss of chyle, which can lead to nutritional depletion and compromise of the immune function [2,4,5,13,17,28].

Failure of conservative therapy, particularly in the setting of sustained high-volume drainage, necessitates escalation of care. This may involve temporary bowel rest supported by parenteral nutrition and, ultimately, targeted procedural solutions. Surgical or minimally invasive approaches, including pleurodesis, thoracic duct ligation, or image-guided lymphatic interventions, are reserved for refractory cases to achieve definitive control of the leak and prevent long-term complications [29,30].

#### 1.4.1. Nutritional and Dietary Interventions

The most important step when managing pediatric chylothorax is to adapt nutrition in such a way that limits chyle production without reducing caloric and protein intake, which are essential for growth and immune function. Long-chain triglycerides (LCTs) need to be restricted because they increase lymphatic flow and should be replaced with medium-chain triglycerides (MCTs), which are absorbed directly into the portal venous system and therefore bypass the lymphatic circulation. In neonates and infants, complete fat exclusion is avoided because lipids are essential for somatic growth and neurodevelopment. Evidence from infants with postoperative chylothorax following cardiac surgery indicates that both centrifuged low-fat breast milk and MCT formulas are effective in reducing chest tube output, without significant differences in their immunological properties [17,31,32,33].

In older children and adolescents, the diet can be restricted to less than 10 g of fat per day or less than 30% of total caloric intake, while adequate protein supplementation is provided [2,4,5,26,27,31].

If there is no response to the adapted enteral nutrition plan and chylous drainage exceeds 20 mL/kg/day, it is recommended to escalate to total parenteral nutrition in order to provide bowel rest. Intravenous lipid emulsions are included to prevent essential fatty acid deficiency while still minimizing lymphatic flow. A prolonged fat restriction, however, needs to be closely monitored both clinically and biochemically in terms of growth and development, immune response and albumin levels, fat-soluble vitamin levels, as well as electrolyte balance [31,34,35].

#### 1.4.2. Pharmacological Therapy

When chylothorax proves refractory to conservative management and pleural fluid accumulation secondary to chyle leakage persists, pharmacological therapy becomes necessary. Octreotide is a long-acting somatostatin analogue that is most commonly used to treat such cases. Its mechanism of action is thought to involve binding to somatostatin receptors located on the vascular and lymphatic endothelium, thereby reducing lymphatic fluid production. This effect is mediated through decreased intestinal fat absorption, reduced splanchnic blood flow, and a subsequent reduction in lymphatic flow through the thoracic duct. Octreotide may be administered either as a continuous intravenous infusion or via subcutaneous injections and is generally well tolerated. The reported adverse effects are typically mild and transient and include most commonly gastrointestinal symptoms, disturbances in glucose metabolism, bradycardia, and temporary elevations of liver enzymes. The optimal dosing regimen of octreotide in children remains controversial, and standardized pediatric guidelines are still lacking, even though it is widely used. Therefore, all available evidence is currently derived mainly from neonatal and small pediatric case series [32,36,37].

Several pediatric studies have reported favorable outcomes with octreotide therapy across different age groups and clinical settings. Roehr et al. [38] reported successful treatment in 25 children using either continuous intravenous infusion (mean dose 68 μg/kg/day) or subcutaneous administration (mean dose 40 μg/kg/day), with treatment durations ranging from 7 to 17 days and only minor side effects [39]. In postoperative chylothorax following congenital heart surgery, somatostatin infusion has also shown high efficacy, with complete resolution observed in neonates and young children treated for 7–32 days, although older children required longer treatment courses. Higher-dose regimens exceeding 10 μg/kg/hour have been reported as effective even in cases of large-volume chylothorax, without significant adverse events [40,41,42]. In a brief review, Helin et al. proposed that octreotide therapy may be more effective when started early and administered at higher doses (approximately 80–100 µg/kg/day), rather than beginning with a low dose followed by gradual escalation [38]. Corticosteroids have been suggested as adjunctive therapy in cases of persistent chylous leakage because of their potential to decrease thoracic duct flow and modulate vascular tone. Dexamethasone has demonstrated favorable tolerability in carefully selected pediatric patients. Overall, current evidence supports octreotide as a safe and effective pharmacological option for pediatric chylothorax, although further studies are needed to establish standardized dosing protocols [33,43,44,45]. Other supportive pharmacological measures are often required, such as diuretics for symptomatic relief, as well as albumin and electrolyte replacement. In cases of prolonged lymphocyte and immunoglobulin loss, immunoglobulin supplementation may be considered to prevent secondary immunodeficiency [2,4,5,13,14,23,26,27,28,43].

#### 1.4.3. Interventional Radiology Procedures

In patients with persistent chylothorax despite adequate conservative and pharmacological therapy, minimally invasive interventional radiology techniques may be considered. These include thoracic duct embolization or lymphatic disruption performed under fluoroscopic or magnetic resonance imaging guidance. Such procedures aim to directly occlude the site of lymphatic leakage and have demonstrated high success rates with significantly lower morbidity compared with open surgical approaches. Consequently, interventional radiology is increasingly regarded as an important therapeutic bridge between conservative management and definitive surgical intervention [45,46,47,48].

#### 1.4.4. Surgical Management

Surgical intervention is recommended only for refractory cases. Thus, in case high-output or prolonged chylous drainage lasting more than two to three weeks, or when ongoing losses result in nutritional deterioration and immunosuppression, such management may be considered. The primary surgical option for thoracic duct ligation is through thoracotomy or by video-assisted thoracoscopic surgery. Additional procedures include pleurodesis, especially in recurrent or idiopathic chylothorax, and pleuroperitoneal shunt placement in selected chronic cases. Surgical treatment is most indicated in postoperative chylothorax and traumatic injury to the thoracic duct when conservative and medical measures fail to achieve resolution [49,50,51].

#### 1.4.5. Multidisciplinary Approach

Successful management of chylothorax requires a coordinated multidisciplinary approach involving pediatricians, surgeons, interventional radiologists, nutrition specialists, and intensive care physicians. Close and continuous monitoring of daily pleural drainage volume, nutritional status, electrolyte balance, and immune function is essential to guide timely escalation of therapy and to prevent metabolic and infectious complications. This integrated strategy plays a critical role in optimizing outcomes and minimizing morbidity in children with chylothorax [2,4,13,32,45].

## 2. Case Report

A 13-year-old patient, born to a non-consanguineous couple, with a current weight of 45 kg, weight-for-height of 56 kg, and a height of 160 cm (50th percentile), height-for-age 159.70 ± 5.9, Ht SDS = −0.05, and BMI (Body Mass Index) of 17.57 kg/m^2^ (25th percentile), has been known since birth to have a congenital heart malformation (severe valvular and supravalvular pulmonary stenosis, severe valvular and supravalvular aortic stenosis, mild aortic regurgitation, and grade I mitral regurgitation). The patient underwent a single open-heart surgical repair via median sternotomy three years ago, in November 2016 (at age 10 years). The procedure consisted of pulmonary artery trunk enlargement/plasty for supravalvular pulmonary stenosis and ascending aortic plasty for severe valvular and supravalvular aortic stenosis. No additional cardiac surgical interventions or catheter-based procedures have been performed since the initial repair.

The patient developed bilateral lower-limb lymphedema at age 5 years, preceding her cardiac surgery by approximately 5 years. There has been no documented history of complications related to the lymphedema. The lymphedema has been managed conservatively and has remained clinically stable without significant progression. She presented to the Emergency Care Unit of the “Louis Țurcanu” Children’s Emergency Hospital with fatigue, decreased exercise tolerance, vertigo, and diaphoresis. In the Emergency Care Unit, the patient was examined and investigated.

The patient remained largely asymptomatic until approximately mid-2020, when she began experiencing insidious onset of fatigue and mildly decreased exercise tolerance. No fever, significant weight loss, chest pain, or acute respiratory distress was reported during this interval. No medical or procedural interventions were undertaken during this period. The patient continued her baseline cardiac medications. Physical activity was progressively limited due to increasing fatigue, though she did not require activity restriction until shortly before hospital admission. A chronological summary of the patient’s clinical course is presented in Table 1.

Upon presentation to the Emergency Care Unit, chest radiography was performed as the initial imaging modality, which demonstrated a large right-sided pleural effusion causing partial lung compression, consistent with a space-occupying pleural process (Figure 1). Following the chest radiograph, computed tomography (CT) of the chest was obtained for further characterization, revealing a right-sided hydropneumothorax with an air layer measuring up to 2 cm, a posterobasal fluid collection, and multiple pleural adhesions extending into the lower two-thirds of the lung with associated partial lung collapse. Notably, no mediastinal masses or lymphadenopathy were identified on CT imaging.

Following confirmation of chylothorax and initiation of treatment, follow-up chest CT was performed during hospitalization to assess therapeutic response, demonstrating marked reduction in the pleural effusion layer (Figure 2). Additionally, chest radiography was repeated before and after clamping of the drainage tube at the end of the 14-day treatment period to confirm resolution prior to drain removal.

The patient was surgically evaluated, and a chest drainage tube connected to a SINAPI closed chest drainage system was placed (Figure 3). The closed chest drainage system used for continuous evacuation of pleural fluid, allowing daily quantification of chylous drainage and monitoring of treatment response. Initially, approximately 700 mL of yellowish-bloody fluid was drained, and intermittent clamping was recommended until complete evacuation of the pleural fluid was achieved.

The currently observed pleural collection accumulated slowly, slowly over approximately 8 months, with its first documentation dating back to December 2019, when echocardiography described a small right-sided pleural effusion. Imaging reassessments performed in March and July 2020 by echocardiography confirmed the progressive accumulation of pleural fluid, and hospitalization was recommended.

At the time of hospital admission, the patient was alert, cooperative, and remained afebrile throughout the initial assessment. She presented with pale skin, bilateral right knee bruises (following a play-related accident), and an old median sternotomy scar (post-cardiac surgery). Nutritional status was fair, with a BMI of 17.57 kg/m^2^ (25th percentile). A right-sided pleural drainage tube was present in the fourth intercostal space (inserted during initial surgical evaluation). Bilateral lower-limb lymphedema, low posterior hairline, broad chest and widely spaced nipples were noted. Vital signs: Oxygen saturation (SaO_2_) was 97–98%, respiratory rate (RR) was 24–26 breaths/min., heart rate (HR) was 62 bpm. and blood pressure was 100/60 mmHg. Chest percussion revealed dullness over the lower one-third of the right lung field. Pulmonary auscultation showed absent breath sounds over the lower one-third of the right lung, with normal breath sounds over the entire left lung and no added crackles. Cardiac auscultation revealed rhythmic and bradycardic heart sounds, a harsh grade III/VI systolic murmur at the left second intercostal space, and a systolic murmur with a diastolic component at the third–fourth left intercostal space along the left sternal border and cardiac apex, with radiation to multiple sites. Bilateral femoral pulses were present. Abdominal examination revealed a soft, non-distended abdomen. The liver edge was palpable 2–3 cm below the right costal margin, with normal parenchymal consistency. No splenomegaly was detected, and there was no tenderness or guarding. Percussion revealed no shifting dullness or fluid thrill to suggest ascites, and bowel sounds were normal on auscultation. Neurological examination was unremarkable, with no focal neurological deficits identified.

On admission, the radiological workup in this case proceeded in two steps: chest radiography (posteroanterior and lateral views) was performed first as the initial modality, demonstrating a massive right-sided pleural effusion with partial lung compression and contralateral mediastinal shift (Figure 1). Computed tomography (CT) of the chest was then obtained 2 days after admission for detailed characterisation, revealing right-sided hydropneumothorax with an air layer of up to 2 cm on lung window imaging, a posterobasal fluid collection, multiple pleural adhesions extending into the lower two-thirds of the lung, and partial lung collapse (Figure 2). During treatment, abdominal ultrasound was performed every five days to monitor for octreotide-related biliary complications. Response was subsequently confirmed on follow-up CT chest and on serial chest radiography performed before and after drain clamping prior to discharge. Follow-up CT chest was performed to objectively confirm the therapeutic response, demonstrating a marked reduction in the pleural effusion layer compared with the admission CT.

This case required multidisciplinary collaboration for management. The patient was clinically monitored, and vital parameters remained within the following ranges: arterial oxygen saturation (SaO_2_) = 99–100%, HR = 45–66 bpm, mean BP = 95/55 mmHg, and RR = 16–24 breaths/min. The functionality and patency of the pleural drainage system, as well as the appearance and volume of the collected fluid, were continuously monitored, with daily recording of the appearance and volume of collected fluid.

During hospitalization, a daily volume ranging from 700 mL to 29 mL of pleural fluid was drained, with an appearance varying from yellowish to serosanguineous. Biochemical analysis of the pleural fluid (cholesterol 1.90 mmol/L, triglycerides 8.59 mmol/L) corroborated corresponding serum values (cholesterol 2.77 mmol/L, triglycerides 0.76 mmol/L), yielding a pleural fluid/serum triglyceride ratio of 11.30 and a pleural fluid/serum cholesterol ratio of 0.68. Macroscopic and microscopic analysis of the pleural fluid revealed a serosanguineous effusion with well-represented cellularity on smear, comprising lymphocytes, polymorphonuclear cells, hyperplastic reactive mesothelial cells, macrophages, and degenerated cells against a sparse haematic background, with overall features consistent with an inflammatory process and a slowly accumulating collection. Cytological analysis of the pleural fluid (protein 41.9 g/L, 79% lymphocytes) confirmed the diagnosis of chylothorax. Notably, adenosine deaminase (ADA) levels in pleural fluid were not measured in this patient.

In parallel with clinical management, extensive investigations were undertaken to determine the etiology of the pleural effusion. Several potential causes were systematically excluded. Hematologic and oncologic disorders were ruled out, as imaging studies (chest computed tomography and abdominal ultrasound) showed no tumoral formations, and laboratory tests (complete blood count, peripheral blood smear, and lactate dehydrogenase) were within normal limits, with no specific abnormalities detected. Ovarian tumors were also excluded. Cancer antigen 125 (CA-125) was slightly elevated in the context of menstruation, while carcinoembryonic antigen (CEA) and beta-Human Chorionic Gonadotropin (β-HCG) values were within normal ranges. Abdominal ultrasound performed during hospitalization did not reveal any pelvic or abdominal masses, ascites, or lymphadenopathy.

An infectious etiology was carefully investigated. Tuberculosis was excluded based on negative bacteriological examination for BK (Koch’s bacillus) in pleural fluid, a negative genetic test for BK, negative cultures for common bacteria, and a negative QuantiFERON-TB Gold test, despite an IDR PPD ((Purified protein derivative (PPD) skin test)) of 10 mm. Pyothorax was also ruled out, as pleural fluid analysis showed only 8% polymorphonuclear cells, and both C-reactive protein and erythrocyte sedimentation rate were within normal ranges. A cardiac cause was considered unlikely. Transudative effusion was excluded by pleural fluid analysis, and ultrasound showed normal inferior vena cava and suprahepatic vein dimensions. In addition, biomarkers of heart failure and cardiac dysfunction were within normal values.

Given the co-existence of congenital heart disease, chronic chylothorax, and bilateral lower-limb lymphedema in a pediatric patient, genetic syndromes were also investigated. A normal karyotype (46,XX) excluded Turner syndrome (complete or partial monosomy X). Although the clinical phenotype raised suspicion of a Noonan-like syndrome, genetic testing through sequencing analysis of a panel of 174 genes did not identify a pathogenic variant in the *CBL* gene. Noonan syndrome can cause both congenital heart disease and chylothorax through primary lymphatic dysplasia. Yellow nail syndrome was also ruled out. Following confirmation of the diagnosis of chylothorax, fat-free oral nutrition was initiated (<0.5 g lipids per serving, using medium-chain triglycerides [MCT]). Prophylactic antibiotic therapy was started with ceftriaxone (2 g/day for 30 days), followed by phenoxymethylpenicillin (3 g/day, equivalent to 3 million IU/day). Given the prolonged presence of a chest drainage tube and the anticipated duration of pleural fluid evacuation, prophylactic antibiotic therapy was initiated to reduce the risk of secondary pleural infection. Although chyle has bacteriostatic properties, prolonged drainage and repeated manipulation of the drainage system may increase infection risk, particularly in pediatric patients with potential nutritional and immunological compromise.

In this patient, the decision was also influenced by the recent history of antibiotic exposure prior to admission (amoxicillin–clavulanate), administered in the context of suspected respiratory infection, which raised concern for partially treated infection and potential bacterial selection. Consequently, broader antimicrobial coverage was chosen initially, in accordance with local institutional practice for prolonged pleural drainage, with close clinical and biological monitoring throughout hospitalization.

Prophylactic antifungal treatment with nystatin (1.5 million IU/day) was added. Anti-inflammatory therapy with dexamethasone (3 mg/kg/day) was administered with gradual dose tapering. Supportive and symptomatic treatments included analgesics, gastric protection, hepatoprotective agents, ursodeoxycholic acid, probiotics, and simethicone. Roborant therapy and parenteral maintenance of nutrition and hydration (~500 mL/day) were also provided.

Baseline cardiac and supportive medication was continued, including bisoprolol (2.5 mg/day with gradual dose reduction and subsequent discontinuation), magnesium lactate dihydrate (500 mg/day), and vitamin D_3_ (1000 IU/day).

To initiate treatment with Sandostatin (octreotide) solution for injection 100 µg/mL, an initial infusion rate of 0.5 µg/kg/h (600 µg/24 h) was established. The drug was diluted in 0.9% NaCl and administered by continuous intravenous infusion at a rate of 2.2 mL/h using an automatic syringe pump, in parallel with a 5% glucose solution (500 mL at a rate of 20 mL/h).

During the first 24 h of octreotide treatment, the patient was monitored in the Intensive Care Unit, with good tolerance and no major adverse events. Octreotide administration was subsequently continued in the Pediatrics III ward. Considering that the daily volume of pleural fluid ranged between 65 and 45 mL, the octreotide infusion rate was gradually increased by 0.5 µg/kg/h every 3 days, up to a maximum dose of 2 µg/kg/h (2000 µg/24 h). At this dose, octreotide was diluted in 0.9% NaCl (5 mL octreotide + 45 mL 0.9% NaCl) and administered by continuous intravenous infusion at a rate of 9 mL/h, for a total treatment duration of 14 days.

Under this treatment regimen, the clinical course was slowly favorable. The daily amount of drained pleural fluid progressively decreased from 700 mL/day to 29 mL/day.

Laboratory monitoring included complete blood counts, plasma glucose, liver function tests, creatinine, bilirubin, cholesterol, and triglyceride levels. Abdominal ultrasound was performed every 5 days to detect possible treatment-related adverse effects, particularly asymptomatic biliary lithiasis. Follow-up chest CT revealed a marked reduction in the pleural effusion layer.

Cardiac evaluation and echocardiography established the following diagnoses: operated supravalvular pulmonary stenosis (pulmonary artery trunk enlargement/plasty in 2016), hemodynamically significant pulmonary insufficiency, grade II residual aortic stenosis (after ascending aortic plasty for severe valvular and supravalvular aortic stenosis), and grade II aortic insufficiency.

At the end of the 14-day treatment period, chest radiography was performed before and after clamping of the drainage tube. Accidental drain tube dislodgement occurred on day 38, for which surgical consultation was requested. Wound suturing was performed, and an occlusive adhesive-absorbent dressing was applied to seal the chest tube removal site, with good local and overall outcomes. Vital signs monitoring continued, and the patient remained asymptomatic.

The patient was discharged without respiratory distress and in hemodynamically stable condition and was referred to a specialized center for lymphangiography and magnetic resonance lymphangiography.

## 3. Discussion

Here we present a case of a child with a first documented pleural effusion on echocardiography, identified approximately three years post-surgery. Several potential etiologies for this patient’s chylothorax that remain to be considered are traumatic causes, malformations of the lymphatic system, and idiopathic chyle leakage. Traumatic mechanisms are plausible in this case, given the patient’s history of cardiac surgery performed three years earlier, which was followed by an episode of pleural effusion one month postoperatively that may have represented an unrecognized chylothorax or a transient postoperative effusion that resolved spontaneously and was unrelated to the current chylothorax. Additionally, the patient experienced a car accident one year prior to admission, which could have contributed to delayed lymphatic injury. This delayed presentation, occurring approximately three years after cardiac surgery, is consistent with late-onset chylothorax, which has been reported to occur months to years following cardiothoracic procedures. Late occurrences of chylothorax after trauma, although very rare, have been described in several patients, mostly adults, and the interval from injury to diagnosis varies significantly from 13 days to 20 years. In the described cases, traumatic chylothorax most commonly resulted from injury to the thoracic duct following thoracic or thoracolumbar spine fractures, blunt chest trauma, or, less frequently, iatrogenic injury during spinal or thoracic surgery [52,53,54,55,56,57,58,59,60,61,62,63,64,65,66,67].

Although our patient did not present with clinical features classically suggestive of ovarian malignancy, the decision to measure cancer antigen 125 (CA-125), carcinoembryonic antigen (CEA), and beta-human chorionic gonadotropin (β-HCG) was guided by several considerations. First, CA-125 is also elevated in serous effusions of various etiologies, including pleural and peritoneal inflammation; second ovarian cancer complicated with chylothorax is a rare condition with a relatively poor prognosis. CEA was measured to help exclude occult epithelial malignancies, which can present with chylothorax through lymphatic obstruction or infiltration even in the absence of overt clinical signs. Third, β-HCG was included to rule out germ cell tumors, which, although rare, represent an important diagnostic consideration in the adolescent age group and may present with pleural effusion as an initial manifestation [67]. Both CEA and β-HCG values were within normal ranges. Taken together, these results effectively excluded an occult malignant etiology for the chylothorax and supported the decision to pursue alternative diagnostic pathways. The exclusion of tuberculosis in this case warrants careful interpretation given the inherent diagnostic limitations of available tests for pleural tuberculosis. In our patient, tuberculosis was investigated through multiple modalities: microscopic examination of the pleural fluid for acid-fast bacilli (Mycobacterium tuberculosis) was negative, genetic testing (PCR) for M. tuberculosis was negative, pleural fluid cultures for common bacteria were negative, and the QuantiFERON-TB Gold test was negative. However, the tuberculin skin test (IDR PPD) measured 10 mm.

The bilateral lower-limb lymphedema suggests an underlying lymphatic drainage disorder, which can be caused by an underlying malformation of the lymphatic system. The association between congenital heart disease, bilateral lower limb lymphedema, and chylothorax has been described in the literature, particularly in the context of genetic syndromes such as Noonan syndrome, although no definitive syndromic diagnosis has been established in our patient. The National Society of Genetic Counselors specifically recommends RASopathy gene panels for patients with valvar pulmonic stenosis combined with other cardiovascular malformations or extracardiac features [68]. Turner syndrome was considered given the female sex, congenital heart disease, and lymphedema. Noonan syndrome was considered because chylothorax is a recognized feature of this condition, and pulmonary valve stenosis is the most common cardiac defect in Noonan syndrome. Genetic testing of a 174-gene panel, including RASopathy-associated genes, did not identify a pathogenic variant. A comprehensive gene panel was selected due to the genetic heterogeneity of lymphatic disorders (involving multiple genes including *PIEZO1*, *CCBE1*, *FAT4*, *ADAMTS3*, *MDFIC*, *CELSR1*, and *FZD6*) and the phenotypic overlap between syndromes [69].

Finally, idiopathic chylothorax cannot be excluded in the absence of a clearly identifiable triggering factor.

Careful attention to clinical information plays a crucial role in establishing the diagnosis. It is essential to recognize that the accumulation of large volumes of chylous effusion may be slow and progressive, allowing pulmonary adaptation, in which case the typical respiratory manifestations of chylothorax may be minimal or even absent, which can delay clinical suspicion. The appearance of chest tube drainage can provide an important clue because a milky-yellow fluid is characteristic; however, in some cases, the effusion may appear serosanguineous or hemorrhagic [2,4,70].

In children, notable cases of late-onset chylothorax that were described involved three-month-old male infant with late-onset congenital diaphragmatic hernia and associated chylothorax, a two-year-old boy with cough-induced chylothorax that debuted 15 days after a heavy paroxysmal coughing episode without fever and a 12-years old boy with chylothorax onset after 3–4 months from a traumatic event—a fall from his bicycle. The first case was resolved by laparoscopic surgery and the other two cases by nutritional and pharmacological therapy with Octreotide [71,72,73,74]. However, year-long intervals remain extremely rare and hard to diagnose. These cases were usually resolved by surgical interventions like thoracoscopic thoracic duct ligation and pleurodesis [52].

To date, most reported cases of octreotide-treated chylothorax involve neonatal populations, especially postoperative congenital heart disease patients. In contrast, pediatric cases beyond infancy remain scarce in the literature, and standardized treatment guidelines for children are still lacking. Consequently, dosing regimens vary considerably between studies, and therapeutic decisions are often based on institutional experience or extrapolation from neonatal data [4,17,36,37,38,39,43,45,49,50]. In the present case, octreotide proved effective at a dose of 0.5 µg/kg/h (600 µg/24 h) administered over 14 days, leading to a gradual and sustained reduction in pleural fluid output.

Several complicating factors continue to challenge the management of chylothorax. These include frequent failure of definitive therapy, heterogeneity in dosing protocols, the absence of consensus guidelines, and the diverse clinical backgrounds of affected patients. Furthermore, prolonged chyle loss exposes patients to risks of malnutrition, electrolyte imbalance, immunosuppression, and infection, emphasizing the importance of early diagnosis and individualized treatment strategies [2,4,28].

This case highlights the importance of considering delayed chylothorax in children with a history of cardiac surgery, even several years after the initial intervention. The coexistence of peripheral lymphedema may provide an important clinical clue suggesting an underlying lymphatic vulnerability. Early recognition and individualized conservative management, including nutritional therapy, drainage, and pharmacologic treatment with octreotide, may lead to favorable outcomes and prevent the need for surgical intervention.

Beyond the rarity of chylothorax itself, several features of this case compounded the diagnostic challenge in ways that deserve explicit discussion. First, the pleural fluid was not classically milky: the initial drainage yielded a yellowish-serosanguineous effusion. The diagnosis was ultimately biochemical, resting on markedly elevated pleural triglycerides (8.59 mmol/L) and a pleural fluid/serum triglyceride ratio of 11.3, combined with lymphocyte predominance (79%) on cytology. Second, the three-year interval between cardiac surgery and diagnosis effectively removed postoperative chylothorax from the front of the differential, particularly because a pleural effusion occurring one month after surgery had resolved without specific treatment. Third, the patient’s presenting symptoms of fatigue, decreased exercise tolerance, vertigo, and diaphoresis were clinically indistinguishable from exacerbation of her underlying congenital heart disease, and preserved oxygen saturation despite a massive effusion further attenuated respiratory suspicion. Fourth, a broad differential had to be systematically excluded before the diagnosis could be accepted: transudative effusion from cardiac failure was excluded by exudate criteria and normal cardiac biomarkers; empyema by the absence of fever, normal inflammatory markers, and negative microbiological studies; pseudochylothorax by a non-elevated pleural cholesterol and a pleural/serum cholesterol ratio below 1.0; and malignant effusion by the absence of radiological masses and normal tumour markers. Finally, concomitant findings—a positive tuberculin skin test (IDR PPD 10 mm) and bilateral lower-limb lymphedema—independently widened the differential and mandated additional investigations, including extensive microbiological TB testing and a 174-gene sequencing panel, before alternative aetiologies could be confidently excluded.

An additional challenge in this case was the absence of access to a specialized lymphangiography center in our country. A systematic imaging workup is essential for identifying the underlying cause of chylothorax and guiding therapeutic decisions. In our patient, the initial radiological evaluation included chest radiography and CT. Although MR lymphangiography was considered for further characterization of the lymphatic pathology, this imaging modality was not available at our center. Consequently, the diagnosis relied on a combination of clinical findings, pleural fluid biochemical and cytological analysis, chest radiography, and computed tomography. Although these methods were sufficient to establish the diagnosis of chylothorax, advanced lymphatic imaging could not be performed locally. The patient was therefore referred to a specialized center for lymphangiography, which represents a valuable diagnostic tool for identifying different types and sites of lymphatic leakage, including thoracic duct disruption and peripheral lymphatic malformations.

Current evidence supports conservative therapy as the first-line approach in pediatric chylothorax and related lymphatic disorders. In this context, continuous somatostatin (octreotide) infusion proved beneficial in our patient, reinforcing its role as an effective adjunct to dietary and drainage measures.

Clinical Lessons

- Late-onset chylothorax may occur several years after cardiac surgery and should be considered in children with unexplained pleural effusion.
- The presence of peripheral lymphedema should raise suspicion of an underlying lymphatic disorder.
- Continuous octreotide infusion, even at relatively moderate doses (0.5–2 µg/kg/h in this case), may lead to progressive reduction in chylous drainage and avoid surgical intervention.
- Successful management requires a multidisciplinary approach involving pediatricians, cardiologists, surgeons, nutrition specialists, and radiologists.

## 4. Conclusions

This case represents a late-onset chylothorax, occurring approximately three years after the initial cardiac surgery. It appropriately illustrates the need for a multidisciplinary approach in the management of chronic chylothorax in childhood because of the diagnostic complexity and because of the therapeutic challenges involved. Pediatric chylothorax remains a rare and challenging condition with heterogeneous etiologies and no standardized treatment protocols. This case highlights the importance of a systematic diagnostic approach based on clinical findings, pleural fluid analysis, and imaging, particularly when advanced lymphatic studies are not immediately available.

Conservative management, including pleural drainage, dietary fat restriction, and continuous octreotide infusion, was effective in achieving a progressive reduction in chylous effusion and avoiding surgical intervention. These findings support the role of octreotide as a useful adjunct in selected pediatric cases.

Further studies are needed to establish standardized diagnostic pathways and to define optimal dosing and duration of pharmacological therapy in children with chylothorax.

## Figures and Tables

**Figure 1:**
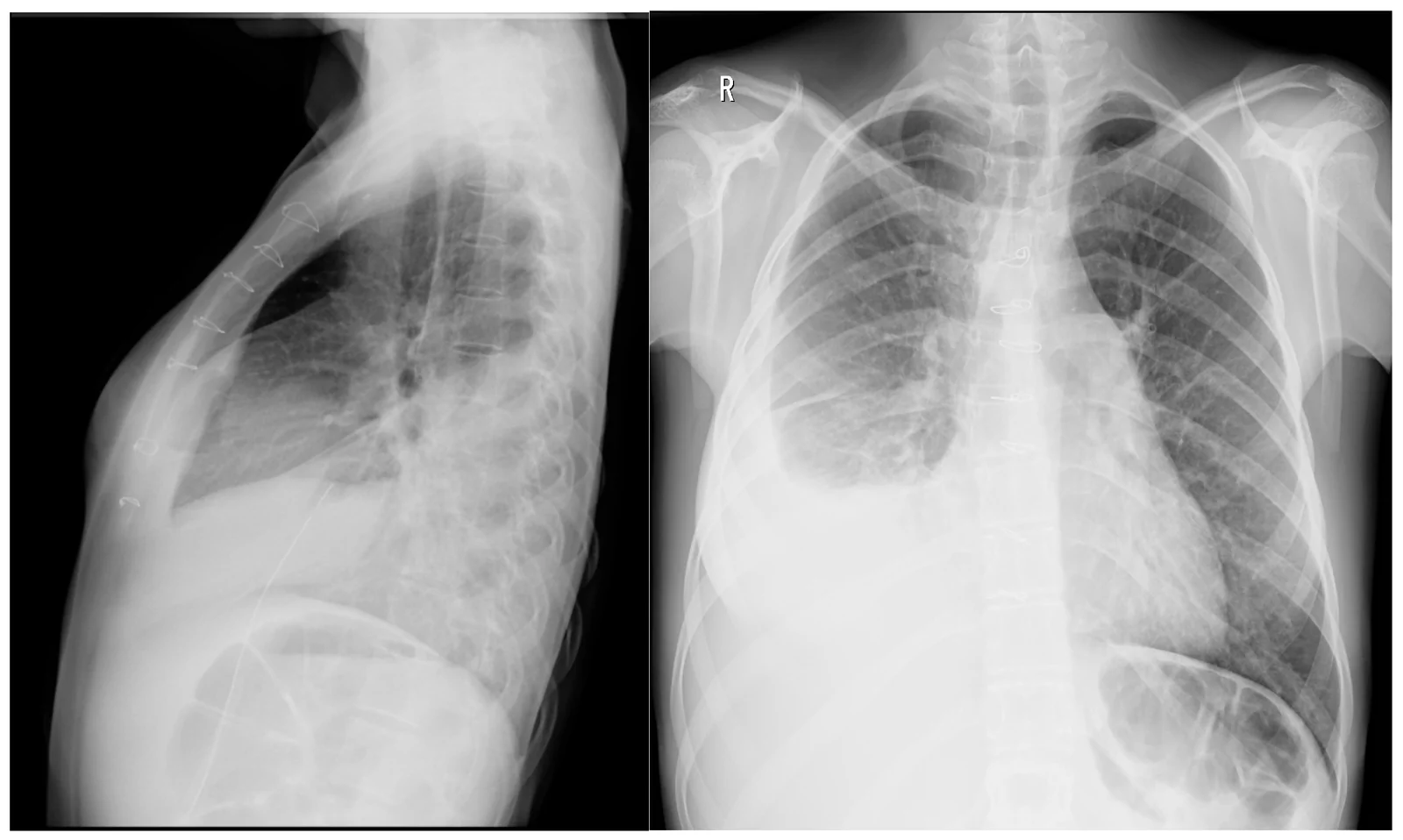
Chest radiograph, lateral and antero-posterior view showing a large right-sided pleural effusion causing partial lung compression, consistent with a space-occupying pleural process.

**Figure 2:**
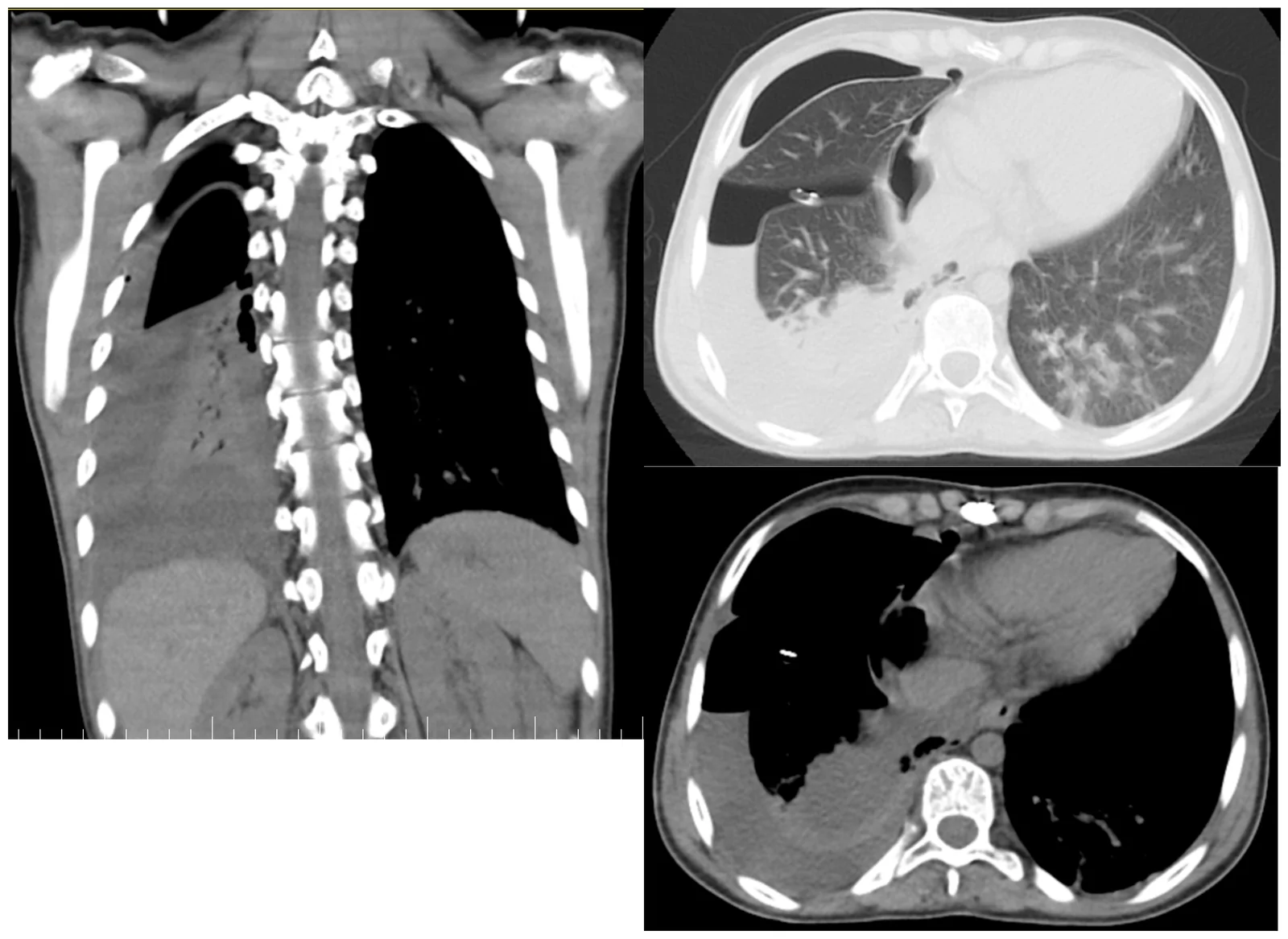
Chest computed tomography showing a right-sided pleural fluid collection with associated pleural adhesions and partial lung collapse, without evidence of mediastinal masses or lymphadenopathy.

**Figure 3:**
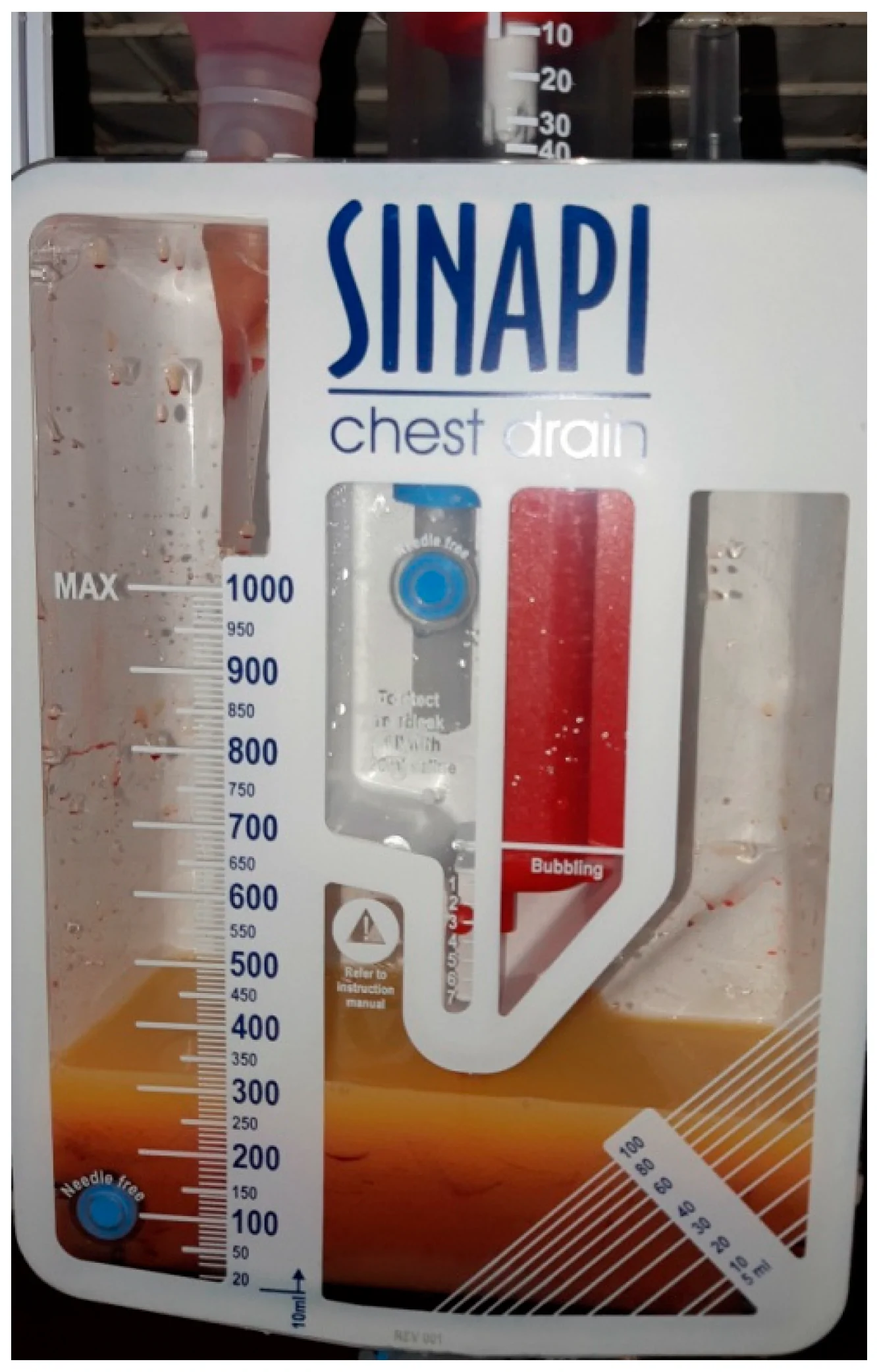
SINAPI chest drain.

**Table 1:** Timeline of the Patient’s Clinical Course.

Time Point	Event
Age 5 years	Onset of lower-limb lymphedema
2016	Cardiac surgery for congenital heart disease
1-month post-surgery	Pleural effusion episode
December 2019	Small pleural effusion first documented
March–July 2020	Progressive effusion on imaging
Admission—July 2020	Massive right-sided chylothorax diagnosed
Day 1	Pleural drainage initiated
Day 2	Fat-restricted diet started
Day 3	Octreotide infusion initiated
Day 3–14	Dose titration and monitoring
Day 14	Marked reduction in drainage
Discharge	Clinical stabilization and referral

## Data Availability

The data presented in this study are available on request from the corresponding author. The data are not publicly available due to privacy restrictions, as they contain sensitive genetic and clinical information that could compromise patient confidentiality.

## Abbreviations

The following abbreviations are used in this manuscript:

CT	computed tomography
MCT	medium-chain triglycerides
Ht SDS	Height Standard Deviation Score
BMI	Body mass index
IDR PPD	Intradermic Reaction to Purified Protein Derivative
BK	Bacillus Koch—Mycobacterium tuberculosis
CBL	Casitas B-lineage Lymphoma proto-oncogene
